# Long-term results of a phase II study of hypofractionated proton therapy for prostate cancer: moderate versus extreme hypofractionation

**DOI:** 10.1186/s13014-019-1210-7

**Published:** 2019-01-10

**Authors:** Boram Ha, Kwan Ho Cho, Kang Hyun Lee, Jae Young Joung, Yeon-Joo Kim, Sung Uk Lee, Hyunjung Kim, Yang-Gun Suh, Sung Ho Moon, Young Kyung Lim, Jong Hwi Jeong, Haksoo Kim, Weon Seo Park, Sun Ho Kim

**Affiliations:** 1 Proton Therapy Center, National Cancer Center, 323 Ilsan-ro, Ilsandong-gu, Goyang-si, Gyeonggi-do 10408 Republic of Korea; 2 Center for Prostate Cancer, National Cancer Center, 323 Ilsan-ro, Ilsandong-gu, Goyang-si, Gyeonggi-do 10408 Republic of Korea; 3 Department of Radiation Oncology, Hallym University Dongtan Sacred Heart Hospital, Seoku-dong, Hwaseong-si, Gyeonggi-do 18450 Republic of Korea

**Keywords:** Dose hypofractionation, Treatment outcome, Prostatic neoplasms, Proton therapy, Radiotherapy

## Abstract

**Background:**

We performed a prospective phase II study to compare acute toxicity among five different hypofractionated schedules using proton therapy. This study was an exploratory analysis to investigate the secondary end-point of biochemical failure-free survival (BCFFS) of patients with long-term follow-up.

**Methods:**

Eighty-two patients with T1-3bN0M0 prostate cancer who had not received androgen-deprivation therapy were randomized to one of five arms: Arm 1, 60 cobalt gray equivalent (CGE)/20 fractions/5 weeks; Arm 2, 54 CGE/15 fractions/5 weeks; Arm 3, 47 CGE/10 fractions/5 weeks; Arm 4, 35 CGE/5 fractions/2.5 weeks; and Arm 5, 35 CGE/5 fractions/4 weeks. In the current exploratory analysis, these ardms were categorized into the moderate hypofractionated (MHF) group (52 patients in Arms 1–3) and the extreme hypofractionated (EHF) group (30 patients in Arms 4–5).

**Results:**

At a median follow-up of 7.5 years (range, 1.3–9.6 years), 7-year BCFFS was 76.2% for the MHF group and 46.2% for the EHF group (*p* = 0.005). The 7-year BCFFS of the MHF and EHF groups were 90.5 and 57.1% in the low-risk group (*p* = 0.154); 83.5 and 42.9% in the intermediate risk group (*p* = 0.018); and 41.7 and 40.0% in the high risk group (*p* = 0.786), respectively. Biochemical failure tended to be a late event with a median time to occurrence of 5 years. Acute GU toxicities were more common in the MHF than the EHF group (85 vs. 57%, *p* = 0.009), but late GI and GU toxicities did not differ between groups.

**Conclusions:**

Our results suggest that the efficacy of EHF is potentially inferior to that of MHF and that further studies are warranted, therefore, to confirm these findings.

**Trial registration:**

This study is registered at ClinicalTrials.gov, no. NCT01709253; registered October 18, 2012; retrospectively registered).

## Background

The α/β ratio for prostate cancer is known to be very low (range, 0.9–2.2 Gy) [1–5]. A lower α/β ratio than that of the surrounding normal tissue has suggested that hypofractionated schedules might increase the therapeutic ratio. We have previously reported the interim results of phase II trial comparing five different hypofractionation dose schedules using proton beam therapy (PBT) [6]. Because of its unique dose-distribution, PBT is better for delivering highly conformal radiation to the prostate while sparing the adjacent rectum and bladder [7], and is considered an effective method to deliver high doses per fraction, although there is no clear evidence that PBT offers a clinical advantage over any other form of definitive radiotherapy. As comparative studies on the efficacy of different hypofractionation dose schedules are lacking, we conducted an exploratory analysis of the phase II trial comparing outcomes between moderate hypofractionation (MHF; fractional dose < 5 Gy) and extreme hypofractionation (EHF; fractional dose ≥5 Gy).

## Methods

### Study design and patient eligibility

This was an exploratory single institution phase II trial comparing five different hypofractionated schedules in males with localized prostate cancer. Patients with biopsy-proven androgen-deprivation therapy (ADT)-naive prostate adenocarcinoma, stage T1-3N0M0 and an Eastern Cooperative Oncology Group performance status of 0–2 were eligible for the trial. Patients were randomly assigned to five different dose schedules using block randomization method. Our institutional review board approved the study protocol. For staging, abdomen and pelvic computerized tomography (CT), prostate magnetic resonance imaging (MRI), and whole-body bone scans were performed in all patients. Positron-emitting tomography (PET)/CT was not routinely performed. Table 1 lists each hypofractionated schedule and equivalent dose using 2 Gy (EQD2) in five arms. The rationale for the dose/fractionation schedule was described in our previous report [6]. Briefly, assuming an α/β ratio of 1.5 Gy for prostate cancer and 3 Gy for normal tissue late toxicities, five hypofractionated schedules with the same biologically equivalent dose of 72 Gy in 2 Gy fractions for a late effect were chosen. Each schedule was adjusted to be delivered < 5 times per week (1–4 times per week) to reduce the acute rectal mucosal toxicity. The effect of repopulation for prostate cancer during treatment was not considered [8].

**Table 1:** Five hypofractionated schedules and equivalent dose in 2-Gy

						EQD2		
		Daily dose (CGE)	Number of fractions	fractions / week	Treatment time (Days)	Prostate cancer (α/β = 1.5 Gy)	Acute toxicity (α/β = 10 Gy)	Late toxicity (α/β = 3 Gy)
Reference dose in 2 Gy/fraction		2	36	5	49	72.0	72.0	72.0
MHF	Arm 1	3	20	4 (Mon. Tue, Thu, Fri)	32	77.1	69.6	72.0
	Arm 2	3.6	15	3 (Mon, Wed, Fri)	32	78.7	63.8	71.3
	Arm 3	4.7	10	2 (Tue, Thu)	30	83.3	58.2	72.4
EHF	Arm 4	7	5	2 (Tue, Thu)	14	85.0	55.5	70.0
	Arm 5	7	5	1 (Wed)	28	85.0	46.3	70.0

*EQD2* equivalent dose in 2-Gy fractions *(see Supplementary Appendix of reference* 6), *CGE* cobalt gray equivalent = proton dose in Gy × 1.1, *MHF* moderate hypofractionation, *EHF* extreme hypofractionation

### PBT

Details of the simulation and treatment planning for PBT at our institution have been previously reported [6, 9]. Briefly, three gold markers were inserted into the prostate and used to verify the exact location of the prostate for each treatment session. A balloon was inserted into the rectum and filled with 100 mL saline for both CT and MRI scans to guide treatment planning. A set of 3-mm-thick contrast CT images and MRI scans was acquired on the same day. The CT and MRI images were fused using a registration algorithm and targets were delineated on the fused images. The clinical target volume (CTV) was defined as the whole prostate plus the proximal 1 cm of seminal vesicles, or whole seminal vesicles in cases of involvement. We did not treat seminal vesicles or regional lymph nodes electively when using hypofractionation due to toxicity concerns. The planning target volume (PTV) was created by adding 1.0 cm to the CTV in all directions except posteriorly, where 0.7 cm was added. The treatments were planned using an Eclipse proton beam planning system (Varian Medical Systems, Palo Alto, CA, USA). An opposing pair of bilateral beams was used and the dose was projected to the 100% isodose line. The plan was normalized so that 95% of the PTV received the prescribed dose. The dose-volume constraints for the organs at risk involved a volume receiving 50 Gy EQD2, which should not have exceeded 30% for the rectum and bladder. During every treatment session, digital orthogonal x-ray images were acquired and transferred to the digital image positioning system. The three-dimensional relative locations of the gold markers in the digital images were then compared with those in the reference images. Any differences between the two images were calculated automatically by the system and the treatment couch was adjusted to eliminate any discrepancies greater than 1 mm.

### ADT

Multimodal therapy consisting of radiation and ADT is the current standard of care for high-risk patients. In this study, 17 high-risk patients who had refused ADT or were unfit for ADT due to other medical co-morbidities such as a history of coronary arterial or cerebro-vascular disease, were included and treated with PBT alone. ADT was allowed as a salvage treatment in cases of biochemical or clinical failure during follow-up.

### Statistical analysis

The primary objective was to compare the acute toxicities and determine the best arm with the lowest toxicities, and the secondary objectives were to compare the biochemical failure-free survival (BCFFS), overall survival (OS), and long-term toxicities [6]. Severe acute toxicity was defined as grade ≥ 2 toxicity. With a 90% probability of selecting the best arm with a 30% difference between the best arm and the other four arms in terms of severe acute toxicity, 18 patients were needed per arm. Assuming a follow-up loss of 5%, 19 patients were needed per arm [10]. Thus, a total of 95 patients were scheduled for enrollment. However, because the interim analyses revealed a higher biochemical failure (BCF) in Arm 5 (35 Gy/5 fractions/once per week for 4 weeks), an accrual to Arm 5 was terminated after the enrollment of 12 patients. Survival was estimated using the Kaplan–Meier method and compared using the log-rank test. A Cox regression hazard model was used for multivariate analysis and significance was determined at *p* < 0.05. Toxicity events were compared using the chi-square and Fisher’s exact tests.

### Data collection and follow-up

BCF was defined as an increase in serum prostate specific antigen (PSA) > 2.0 ng/mL from the nadir according to the Phoenix definition (RTOG-ASTRO Phoenix Consensus Conference 2006). Acute gastrointestinal (GI) and genitourinary (GU) toxicities were rated according to the National Cancer Institute Common Toxicity Criteria (https://ctep.cancer.gov/protocolDevelopment/electronic_applications/docs/ctcaev3.pdf) and were assessed weekly during and 1 month after completion of the PBT. Patients were followed every 3 months during the first 2 years, every 6 months during the next 3 years, and annually thereafter. The late GI and GU toxicities were evaluated using the RTOG/European Organization for Research and Treatment of Cancer late radiation morbidity scoring system. The risk groups were divided according to the National Comprehensive Cancer Network Practice Guideline (NCCN V2 2009).

## Results

### Study population

We defined MHF as a fractional dose < 5 Gy per fraction and EHF as a fractional dose > 5 Gy. Of a total of 82 patients, 52 patients were treated with MHF (19 in Arm 1, 16 in Arm 2, and 17 in Arm 3) and 30 were with EHF (18 in Arm 4 and 12 in Arm 5). The baseline patient and tumor characteristics are summarized in Table 2. The median age of the entire study population was 68 years (range, 44–85 years). The majority of patients had Gleason score 6 disease (63%), a PSA < 10 ng/mL (67%), and T1 (35%) or T2 disease (54%). Most of the patients had low (34%) or intermediate (45%) risk disease and 21% had high risk disease.

**Table 2:** Patients’ characteristics

Characteristics	MFH (*n* = 52)	EHF (*n* = 30)	*P*	Total (*n* = 82)
Age, median (range)	68 (44–85)	68 (46–80)	.464	68 (44–85)
ECOG PS				
0	10 (19%)	10 (33%)	.249^a)^	20 (24%)
1	41 (79%)	20 (67%)		61 (75%)
2	1 (2%)	0		1 (1%)
Gleason score				
≤ 6	35 (67%)	17 (57%)	.544^a)^	52 (63%)
7	14 (27%)	10 (33%)		24 (29%)
8–10	3 (6%)	3 (10%)		6 (7%)
Initial PSA (ng/ml)				
< 10	36 (69%)	19 (63%)	.203^a)^	55 (67%)
10–20	12 (23%)	11 (37%)		23 (28%)
> 20	4 (8%)	0 (0%)		4 (5%)
T stage				
T1	20 (38%)	9 (30%)	.362^b)^	29 (35%)
T2	25 (48%)	19 (63%)		44 (54%)
T3	7 (13%)	2 (7%)		9 (11%)
NCCN Risk group				
Low	21 (40%)	7 (23%)	.131^b)^	28 (34%)
Intermediate	19 (37%)	18 (60%)		37 (45%)
High	12 (23%)	5 (17%)		17 (21%)

*MHF* moderate hypofractionation, *EHF* extreme hypofractionation, *ECOG* Eastern Cooperative Oncology Group, *PS* performance status, *NCCN* National Comprehensive Cancer Network V2 2009

^a)^Fisher’s exact test; ^b)^ Chi-square test

### BCFFS and OS

At a median follow-up of 7.5 years (range, 1.3–9.6 years), 35 patients had BCF with 15 in the MHF group and 20 in the EHF group. Among the 15 patients with BCF in the MHF group, three patients eventually developed local recurrences and one patient developed distant metastasis. In the EHF group, there were two local recurrences and one distant metastasis among the 20 patients with BCF.

**Fig. 1:**
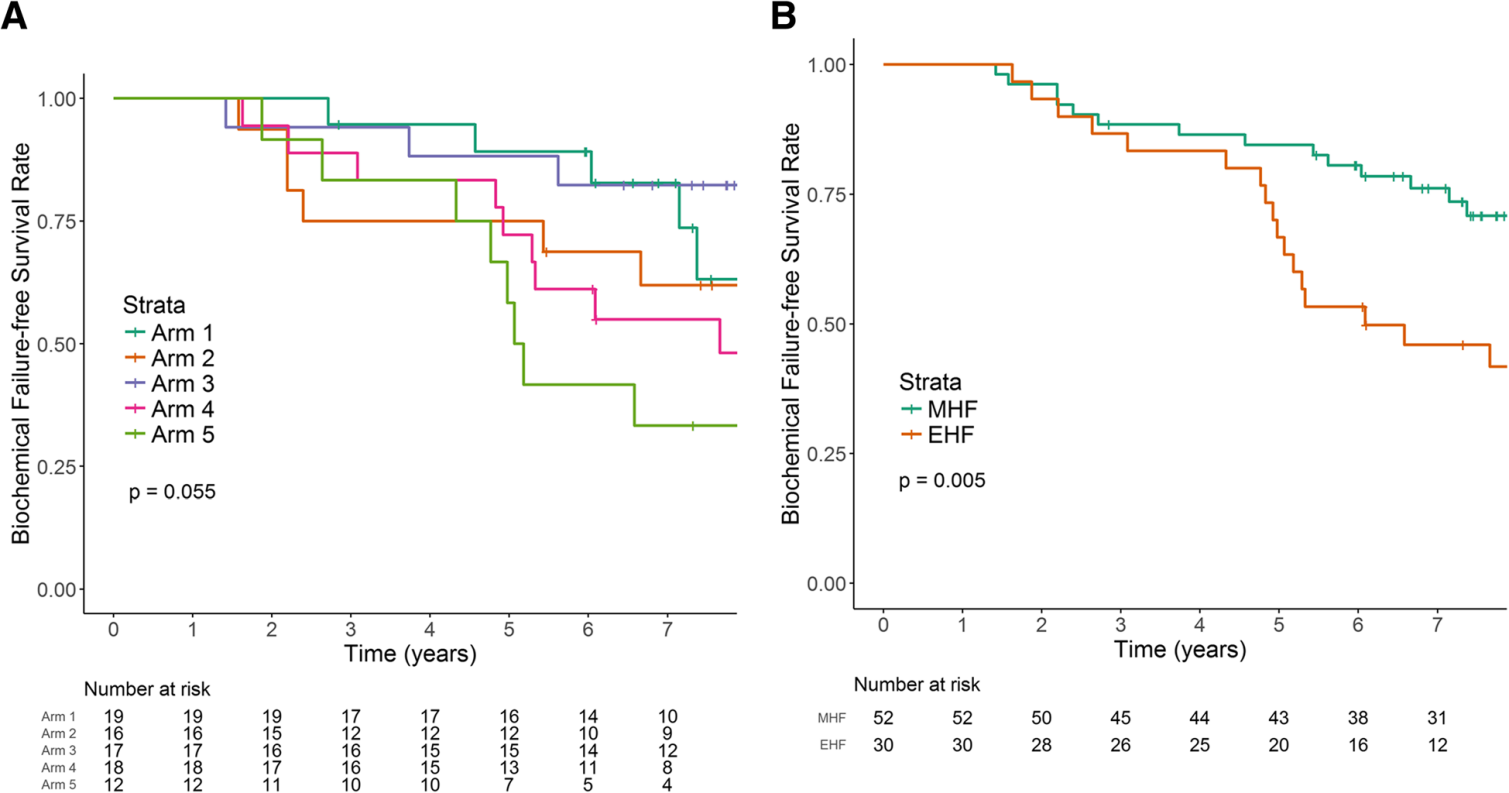
**a** Estimated biochemical failure-free survival (BCFFS) of the original five different dose schedules. **b** Estimated BCFFS after separation into the moderate hypofractionation (MHF) group with a fractional dose < 5 Gy and extreme hypofractionation (EHF) group with a fractional dose ≥5 Gy

**Fig. 2:**
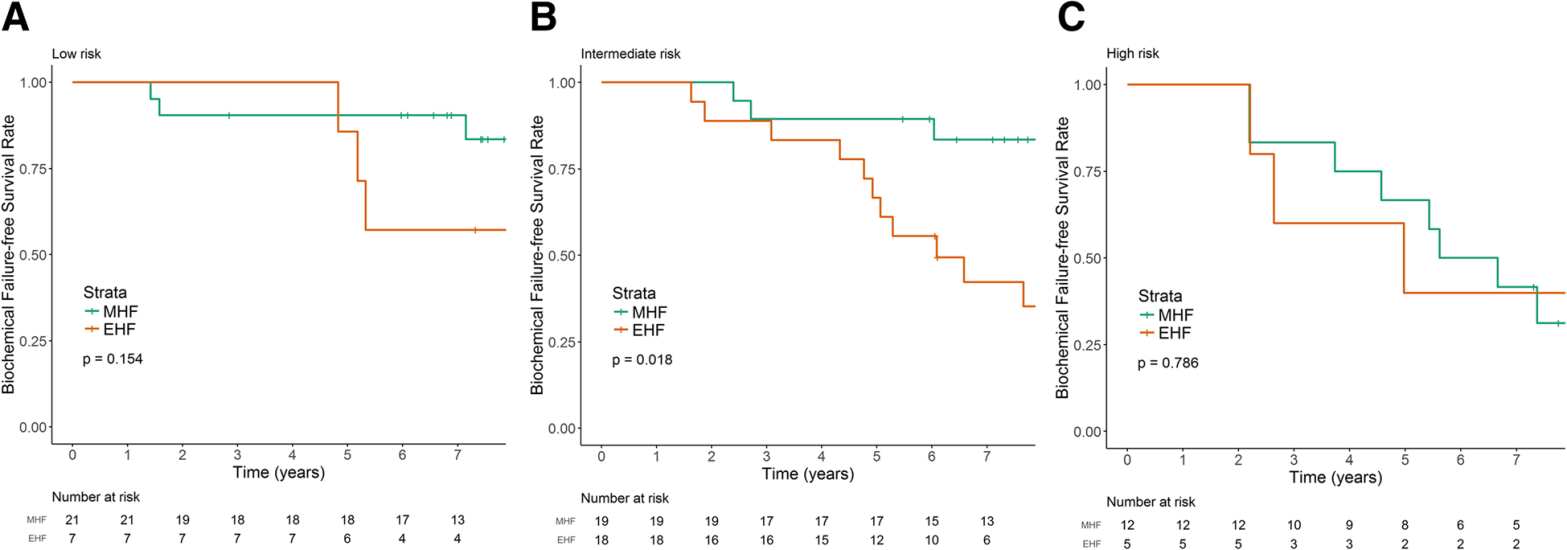
Subgroup analysis of biochemical failure-free survival (BCFFS) according to the NCCN risk groups. **a** Low risk group. **b** Intermediate risk group. **c** High risk group. MHF, moderate hypofractionation; EHF, extreme hypofractionation

The median time to onset of BCF was 5.0 years (range, 1.4–9.1 years). Figure 1 shows the Kaplan–Meier survival curves for the BCFFS in the original five different dose schedules (a) and the MHF and EHF groups (b). A steep decline in the BCFFS in the EHF group was observed after 4 years, which resulted in a significantly lower 7-year BCFFS in the EHF group (46.2 vs. 76.2%; *p* = 0.005). Using multivariable analyses, the hazard ratio was 3.24 for the EHF group (95% confidence interval, 1.51–6.93, *p* = 0.003), after adjusting for age, Gleason score, pretreatment PSA, and T stage. The 7-year BCFFS in the MHF and EHF groups were 90.5 and 57.1% for the low risk group (*p* = 0.154), 83.5 and 42.9% for the intermediate risk group (*p* = 0.018), and 41.7 and 40.0% for the high risk group, respectively (*p* = 0.786) (Fig. 2).

Among the three deaths at the time of analysis, only one was caused by progression of bone metastases from the prostate cancer; the others were caused by other malignancies, including lung cancer and leukemia. The 7-year OS was 97.5% for the entire study population. The OS was not compared between the two groups because of the low frequency of events.

### Acute and late GI and GU toxicities

Table 3 summarizes the acute and late toxicities. No significant differences were observed in the overall incidences of acute and late toxicities. Grade 2 or higher acute GI toxicities were not observed. The incidences of grade 1 and 2 acute GU toxicities were 81 and 4% in the MHF group and 50 and 7% in the EHF group, respectively (*p* = 0.009), and grade 3 acute GU toxicity was not observed in either group. Grade 2 and 3 late GI toxicities were observed in eight (15%) and two (4%) patients in the MHF group and four (13%) and zero patients in the EHF group (*p* = 0.891). Grade 2 late GU toxicities were observed in six (12%) and two (7%) patients in the MHF and EHF groups, respectively (*p* = 0.835).

**Table 3:** Maximum acute and late GI and GU toxicity

Maximum grade	MHF (n = 52)	EHF (n = 30)	*P*	Total (n = 82)
Acute toxicity				
GI				
0	46 (89%)	24 (80%)	.341^b)^	70 (85%)
1	6 (11%)	6 (20%)		12 (15%)
GU				
0	8 (15%)	13 (43%)	.009^b)^	21 (26%)
1	42 (81%)	15 (50%)		57 (70%)
2	2 (4%)	2 (7%)		4 (5%)
Late toxicity				
GI				
0	15 (29%)	10 (33%)	.891^b)^	25 (31%)
1	27 (52%)	16 (53%)		43 (52%)
2	8 (15%)	4 (13%)		12 (15%)
3	2 (4%)	0 (0%)		2 (2%)
GU^a)^				
0	32 (62%)	19 (63%)	.835^a)^	51 (62%)
1	14 (27%)	9 (30%)		23 (28%)
2	6 (12%)	2 (7%)		8 (10%)

*MHF* moderate hypofractionation, *EHF* extreme hypofractionation, *GI* gastrointestinal, *GU* genitourinary

^a)^Chi-square test

^b)^Fisher’s exact test

## Discussion

To our knowledge, this is the first prospective study to compare the efficacy of EHF and MHF in patients with prostate cancer. In our exploratory analyses, the 7-year BCFFS rates of the MHF and EHF groups were 76 and 46%, respectively, for all patients (*p* = 0.005). The effect of the fractionation schedule was statistically significant for the intermediate risk group (84 vs. 43%, respectively; *p* = 0.018), but not for the low or high risk group. For the low risk group, the difference in the 7-year BCFFS was substantial (91 vs. 57%, *p* = 0.154), but did not reach significance because of the small sample size (*n* = 28 patients). For the high risk group, the 7-year BCFFS was suboptimal regardless of the fractionation scheme in which the ADT should have been added to the radiotherapy as standard care. The BCF tended to be a late event with a median time to occurrence of 60.0 months. As shown in Fig. 1, there was a steep decline in the BCFFS in the EHF group between the 4th and 5th years, which further suggested that a long-term follow-up was needed.

There are several possible explanations why the BCFFS of EHF was inferior to that of MHF. The two assumptions in this study were that the α/β ratio was 1.5 Gy and that repopulation was negligible during treatment. Assuming an α/β ratio of 1.5 Gy, the EQD2 for the EHF group was 85 Gy, which was greater than the 77.1–83.3 Gy of the MHF group (Table 1), but the actual outcomes were worse in the EHF group, which was contrary to our expectation. Although most of the evidence supports a very low α/β value for prostate cancer involving < 2.0 Gy [1–4], there have been a few studies supporting higher values. Miralbell et al. [5] reported that the α/β ratio could be increased from 1.5 Gy to 4 Gy when the overall treatment time is longer than the lag period for accelerated repopulation. Williams et al. [11] examined the effects of fraction size and total dose of radiotherapy in 3756 patients treated with radiation alone at three institutions, and estimated an α/β ratio of 3.7 Gy. Nahum et al. [12] reported α/β ratios of 8.5 Gy and 15.5 Gy, when considering the heterogeneity of prostate cancer and hypoxia.

In a review of comparative randomized trials, Pollack et al. [13] reported the results of a trial comparing hypofractionation (HF) (70.2 Gy/26 fractions) with conventional fractionation (CF) (76 Gy/36 fractions). Assuming an α/β ratio of 1.5 Gy, the EQD2 for HF of 84.4 Gy would significantly reduce the biochemical/clinical disease failure (BCDF), but there was no significant difference in the BCDF between the treatment arms (23.3 vs. 21.4% at 5 years, respectively; *p* = 0.268). In the RTOG 0415 trial [14], 1092 men with low risk prostate cancer were randomly assigned to CF (73.8 Gy/41 fractions/8.2 weeks) or HF (70 Gy/28 fractions/5.6 weeks). The 5-year disease-free survival was 85.3 vs. 86.3%, respectively, so it was concluded that the efficacy of the 70 Gy/28 fractions was not inferior to the 73.8 Gy/41 fractions. Incrocci et al. [15] reported the results of a randomized trial (HYPRO) comparing a HF of 64.6 Gy/19 fractions with a CF of 78.0 Gy/39 fractions in patients with intermediate and high risk prostate cancers. Based on an α/β ratio of 1.5 Gy, the EQD2 was 90.4 Gy for the HF compared with 78.0 Gy for the CF. Two-thirds of the patients also received concomitant ADT for 32 months. With a median follow-up of 60 months, the 5-year relapse-free survival was 80.5 vs. 77.1%, respectively (*p* = 0.36). The CHHiP trial [16] was a phase III non-inferiority trial involving patients with localized prostate cancer who were randomly assigned (1:1:1) to the CF arm (74 Gy/37 fractions/7.4 weeks) or to one of two HF arms (60 Gy/20 fractions/4 weeks or 57 Gy/19 fractions/3.8 weeks). Most patients were treated with radiotherapy with 3–6 months of neoadjuvant and concurrent ADT. With a median follow-up of 62.4 months, the BCDF at 5 years was 88.3% in the 74 Gy group, 90.6% in the 60 Gy group, and 85.9% in the 57 Gy group. The 60 Gy group was non-inferior to the 74 Gy group but there was no non-inferiority for the 57 Gy group compared with the 74 Gy group. In the PROFIT trial [17], more than 1200 intermediate risk patients were randomly allocated to a CF of 78 Gy/39 fractions/8 weeks or to a HF of 60 Gy/20 fractions/4 weeks. ADT was not permitted. With a median follow-up of 6.0 years, the 5-year BCFFS was 85% in both arms. All of these studies reported that the α/β ratio of prostate cancer was low, but there was considerable uncertainty concerning the estimated value. Table 4 summarizes the treatment outcomes of the MHF and EHF trials with an estimated EQD2. We showed EQD2 assuming an α/β ratio of 1.5 Gy, as well as 3–4 Gy for comparison. Interestingly, the outcomes between the CF and HF in these trials could be better explained with a higher α/β ratio of 3 or 4 Gy for the first four trials [13–16] except for the PROFIT trial [17]. Likewise, for the present study, assuming an α/β ratio of 3–4 Gy better fitted for the outcomes which were worse among the EHF group. The EQD2s for the EHF group were greater than those for the MHF group when an α/β ratio of 1.5 Gy was assumed (85 Gy vs. 77.1–83.3 Gy). Assuming an α/β ratio of 4 Gy, the EQD2s for each schedule were 70.0, 68.4, 68.2, 64.2, and 64.2 Gy, respectively (Table 4).

**Table 4:** Comparison with other studies

Study	Dose (Gy)/fx	EQD2 (Gy)			OTT (weeks)	No. of patients	Median FU	Risk group (%) (ADT)			RT methods	BCFFS/DFS (%)					Grade ≥ 2 toxicity (%)			
																	Acute		Late	
		a/b = 1.5 Gy	a/b = 3 Gy	a/b = 4 Gy				Low	Intermediate	High		Low	Intermediate	High	All	*P=*	GI	GU	GI	GU
Randomized controlled trials: MHF vs. conventional fractionation																				
Pollack et al. [13]	76.0/38 70.2/26	76.0 84.4	76.0 80.0	76.0 78.4	7.6 5.2	152 152	68 mo	0	66 (20%, 4 mo)	34 (100%, 24 mo)	IMRT	at 5-years	80.5/85.0 82.2/73.8 (no ADT/ADT)	73.9 67.9	78.6 76.7	*.268*	22.5 18.1	14.6 15.3	Not reported	13.4 21.5
RTOG 0415 [14]	73.8/41 70.0/28	69.6 80.0	70.8 77.0	71.3 75.8	8.2 5.6	542 550	70 mo	100 (no ADT)	0	0	3D-CRT/ IMRT				85.3 86.3	*NI* ^a^	10.3 10.7	27.2 27.0	13.9 22.2	22.5 29.5
HYPRO [15]	78.0/39 64.6/19	78.0 90.4	78.0 82.7	78.0 79.7	7.8 6.5	397 407	60 mo	0	27 (67%, 34 mo)	73	IMRT				77.1 80.5	*.36*	31.2 42.0	57.8 60.5	17.7 21.9	19.0 12.9
CHHiP [16]	74.0/37 60.0/20 57.0/19	74.0 77.1 73.3	74.0 72.0 68.4	74.0 70.0 66.5	7.4 4.0 3.8	1065 1074 1077	62 mo	15	73 (97%, 3–6 mo)	12	IMRT	96.7 96.6 90.9	86.8 90.2 86.0	86.5 84.2 78.3	88.3 90.6 85.9	*NI* ^a^	25 38 38	46 49 46	13.7 11.9 11.3	9.1 11.7 6.6
PROFIT [17]	78.0/39 60.0/20	78.0 77.1	78.0 72.0	78.0 70.0	7.8 4.0	598 608	72 mo	0	100 (no ADT)	0	3D-CRT/ IMRT				85.0 85.0	*.16*	10.4 16.3	30.6 30.4	13.9 9.9	22.4 22.0
EHF single arm studies																				
Katz and Kang [22]	35.0–36.25/5	85.0–90.6	70.0–74.3	64.2–68.0	1.0 given daily	515	72 mo	63 (8%)	30 (16%)	7 (55%)	SBRT	at 7-years 95.6	89.6	68.5			4	4	4	9.1
King et al. [19]	35.0–36.25/5	85.0–90.6	70.0–74.3	64.2–68.0	1.0–1.5	1100	36 mo	58 (8%, 3mo)	30 (15%, 4 mo)	11 (38%, 4mo)	SBRT	at 5-years 95	84	81			Not assessed			
Loblaw et al. [18]	35.0/5^b^	85.0	70.0	64.2	4.0	84	55 mo	100 (no ADT)	0	0	SBRT	98	–	–			10	20	8	5
Current trial: prospective phase II study comparing MHF vs. EHF																				
Current trial	MHF	77.1–83.3	71.3–72.4	68.2–70.0	4.4–4.7	52	90 mo	34	45 (no ADT)	21	PBT	at 7-years 90.5	83.5	41.7	76.2	*.005*	0	4	19	12
	EHF	85.0	70.0	64.2	2.0–4.0	30						57.1	42.9	40.0	46.2		0	7	13	7

^a^*EQD2* equivalent dose in 2-Gy (see Supplementary Appendix of reference 6), *OTT* overall treatment time, *FU* follow-up, *ADT* androgen deprivation therapy (% of patients received ADT, duration in months), *RT* radiotherapy, *BCFFS* biochemical failure-free survival, *DFS* disease-free survival, *MHF* moderate hypofractionation, *EHF* extreme hypofractionation, *GI* gastrointestinal, *GU* genitourinary, *IMRT* intensity-modulated radiotherapy, *3D-CRT* 3-dimensional conformal radiotherapy, *SBRT* stereotactic body radiotherapy, *PBT* proton beam therapy, *NI* non-inferior, ^b^Treated once a week

Several single-arm EHF trials using stereotactic body radiotherapy (SBRT) for prostate cancer [18–22] have been conducted to date. The largest series with the longest follow-up [22] involved 515 patients treated with 35–36.25 Gy/5 fractions using SBRT. At a median follow-up of 72 months, the 7-year BCFFSs were 95.8, 89.3, and 68.5% for the low-, intermediate-, and high-risk groups, respectively. In a pooled analysis of 1100 patients from eight centers, King et al. [19] reported 5-year BCFFSs of 95.8, 89.3, and 68.5%, respectively, with a median follow-up of 36 months. The corresponding values determined in our study were 57.1, 42.9, and 40.0% at 7 years, despite a very similar dose fractionation scheme (35 Gy/5 fractions). The superior outcome of the former studies could be attributed to the administration of ADT; 8, 16.3, and 55.3% for the low, intermediate, and high risk groups, respectively, in one study [22], and 8, 15, and 38%, respectively, in the other [19]. In our study, ADT was not administered unless there was BCF during follow-up. Additionally, our study had the longest follow-up with a median of 90 months, which could be attributed to the lower BCFFS rates, because BCF is a late event with a median time to failure of 60 months, which could have been even longer when ADT was administered in the former studies.

Time/dose/fractionation schemes might also have been responsible for the discrepancies in the outcomes. The only noticeable difference in the time/dose/fractionation was the frequency of treatment. In previous studies, treatments were administered daily or every other day with an overall treatment time (OTT) per 1.0–1.5 weeks. In our study, patients were treated twice a week (Arm 4) or weekly (Arm 5) with an OTT of 2–4 weeks. Tumor cell repopulation in prostate cancer has been considered negligible when the OTT is no longer than 9–10 weeks [8]. A recent study [23] estimated the tumor repopulation rate and its onset time from previous reports of prostate cancer [8, 24, 25] based on a linear quadratic model and suggested an onset time of 34 days and not longer than 58 days. A few studies [26–28] have reported a detrimental effect of prolonged OTT in a CF scheme. In a study of 1796 patients with low risk prostate cancer treated with radiotherapy alone, D’Ambrosio et al. [28] reported that prolonged OTT due to treatment breaks was an adverse factor for the BCF. In another study by Thames et al. [26], a multi-institutional retrospective analysis of 4839 patients with low and intermediate risk disease, the OTT and dose were significant predictors of BCFFS, in which OTT was significant in patients treated with ≥70 Gy. However, there was no evidence that an OTT of 2.0–4.0 weeks (< 35 days) negatively affected outcomes when compared with an ultra-short OTT of 1.0–1.5 weeks. An ongoing phase II trial of prostate SBRT called PATRIOT (NCT01423474) which compared 40 Gy/ 5 fractions delivered every other day over 11 days vs. once per week over 29 days is expected to clarify this issue.

Acute GU toxicities were more common in the MHF than the EHF group (85% vs. 57%, *p* = 0.009), but late GI and GU toxicities were not different between the groups. These findings were consistent with EQD2 estimates for acute response (α/β = 10 Gy) and late response (α/β = 3 Gy) in the normal tissue (Table 1).

The strength of the present study includes a long-term median follow-up of 90 months, which showed a split of the survival curve after 4 years. A shorter follow-up would not have detected this discrepancy. Additionally, strict eligibility criteria of the ADT-naive patients revealed the sole effect of the fractionation scheme excluding confounding effects due to ADT.

A limitation of this study is that it was an exploratory analysis with a small sample size, which was originally intended to compare acute toxicity among five different dose schedules. The criteria for dividing these five groups into MHF and EHF groups were therefore somewhat arbitrary, although the same subdivision was recently described as “moderate hypofractionation” and “ultrahypofractionation” [29]. In addition, the risk groups were not evenly distributed between the MHF and EHF groups. There were more intermediate-risk patients in the EHF group and more low- and high-risk patients in the MHF group. To overcome this limitation, we compared BCFFS between the two groups according to the risk groups (Fig. 2) and found a statistically significant difference in the intermediate-risk group despite a small sample size (*p* = 0.018). Although our data showed a substantial difference in the efficacy of different hypofractionation schemes in favor of MHF, the result should be interpreted with caution because it is based on an exploratory analysis with a small sample size; our findings should therefore be confirmed in a larger scaled prospective trial. Treating patients with high-risk disease with PBT alone was suboptimal for the current standard of care, with a 7-year BCFFS of approximately 40% in both the MHF and EHF groups. We did not treat seminal vesicles or regional lymph nodes electively due to toxicity concerns when using hypofractionation. However, we recommend androgen deprivation therapy immediately after the occurrence of biochemical failure. Overall survival at 7 years was 97.5% for all patients with a very low disease-specific mortality of 1.2% at 7 years. Only one among the three deaths was related to the progression of bone metastases from prostate cancer.

## Conclusion

Although we are not able to draw a definitive conclusion from the current exploratory analysis, the efficacy of EHF is potentially inferior to MHF; further studies are therefore warranted to confirm these findings. This study is hypothesis-generating; therefore, radiobiological models must be better understood to design a dose/fractionation study. The comparative efficacy of MFH vs. EHF can only be determined by well-designed phase 3 randomized trials comparing “apples with apples”.

## Abbreviations

ADT: Androgen-deprivation therapy
BCDF: Biochemical/clinical disease failure
BCF: Biochemical failure
BCFFS: Biochemical failure-free survival
CF: Conventional fractionation
CTV: Clinical target volume
EHF: Extreme hypofractionation
EQD2: Equivalent dose using 2 Gy
GI: Gastrointestinal
GU: Genitourinary
HF: Hypofractionation
MHF: Moderate hypofractionation
OS: Overall survival
OTT: Overall treatment time
PBT: Proton beam therapy
PTV: Planning target volume
SBRT: Stereotactic body radiotherapy
